# Autonomic Nervous System Responses to Concussion: Arterial Pulse Contour Analysis

**DOI:** 10.3389/fneur.2016.00013

**Published:** 2016-02-17

**Authors:** Michael F. La Fountaine, Michita Toda, Anthony J. Testa, Vicci Hill-Lombardi

**Affiliations:** ^1^School of Health and Medical Sciences, Seton Hall University, South Orange, NJ, USA; ^2^The Institute for Advanced Study of Rehabilitation and Sports Science, Seton Hall University, South Orange, NJ, USA; ^3^Department of Athletics, University of Wisconsin-Madison, Madison, WI, USA; ^4^Department of Athletics, Seton Hall University, South Orange, NJ, USA

**Keywords:** concussion, sympathetic nervous system, arterial stiffness, exercise intolerance

## Abstract

The arterial pulse wave (APW) has a distinct morphology whose contours reflect dynamics in cardiac function and peripheral vascular tone as a result of sympathetic nervous system (SNS) control. With a transition from rest to increased metabolic demand, the expected augmentation of SNS outflow will not only affect arterial blood pressure and heart rate but it will also induce changes to the contours of the APW. Following a sports concussion, a transient state cardiovascular autonomic dysfunction is present. How this state affects the APW has yet to be described. A prospective, parallel-group study on cardiovascular autonomic control (i.e., digital electrocardiogram and continuous beat-to-beat blood pressure) was performed in the seated upright position in 10 athletes with concussion and 7 non-injured control athletes. Changes in APW were compared at rest and during the first 60 s (F60) of an isometric handgrip test (IHGT) in concussed athletes and non-injured controls within 48 h and 1 week of injury. The concussion group was further separated by the length of time until they were permitted to return to play (RTP > 1week; RTP ≤ 1week). SysSlope, an indirect measurement of stroke volume, was significantly lower in the concussion group at rest and during F60 at 48 h and 1week; a paradoxical decline in SysSlope occurred at each visit during the transition from rest to IHGT F60. The RTP > 1week group had lower SysSlope (405 ± 200; 420 ± 88; 454 ± 236 mmHg/s, respectively) at rest 48 h compared to the RTP ≤ 1week and controls. Similarly at 48 h rest, several measurements of arterial stiffness were abnormal in RTP > 1week compared to RTP ≤ 1week and controls: peak-to-notch latency (0.12 ± 0.04; 0.16 ± 0.02; 0.17 ± 0.05, respectively), notch relative amplitude (0.70 ± 0.03; 0.71 ± 0.04; 0.66 ± 0.14, respectively), and stiffness index (6.4 ± 0.2; 5.7 ± 0.4; 5.8 ± 0.5, respectively). Use of APW revealed that concussed athletes have a transient increase in peripheral artery stiffness, which may be a compensatory adaptation to a paradoxical decline of stroke volume during the transition from rest to a state of increased metabolic demand within 48 h of concussion. This dysfunction of the SNS appeared to be more pronounced among concussed athletes who were removed from participation for >1 week compared to those who resumed play within 7 days.

## Introduction

In 2001, the 1st International Conference on Concussion in Sport (CISG) defined concussion as “a complex pathophysiological process affecting the brain, induced by traumatic biomechanical forces” (1). The concussive injury is caused by impacts to the body (including head) with their impulsive forces being transmitted to the head resulting in short-lived, and in some cases persistent symptoms emanating from a functional disturbance of cortical function resulting in psychological, vestibular, and somatic symptoms (2). Contemporary evidence suggests that the average time-course for the resolution of most symptoms is 7–10 days in 80–90% of cases (2).

The most recent guidelines for the safe return to play (RTP) from concussion provided by the National Athletic Trainers Association (3) suggest that physical activity be reintroduced in a systematic manner such that the intensity and complexity of exertion be progressively increased toward expected levels of the respective sport performed. The limiting feature to progression toward RTP is the re-emergence of concussive symptoms, or evidence of reduced performance from a prior assessment. In other words, if a post-concussion athlete is intolerant to exercise (i.e., exertion above rest), then they have not sufficiently recovered from the injury to be able to engage in sporting activities.

Exercise intolerance following concussion has been investigated as both a consequence of injury and a marker for RTP; in addition to symptoms, intolerance is often demonstrated by the presence of abnormal heart rates (HRs) or blood pressures relative to appropriately matched control subjects for a given workload, and may include reduced HR variability and complexity (4–6). Although these studies demonstrate anomalous or inconsistent performance with physical exertion following concussion, there is no evidence as to what physiological mechanism(s) may be contributing to this transient state of intolerance among individuals who were recently engaging in high levels of physical performance. From a mechanistic perspective, the role of the autonomic nervous system (ANS) and, in particular, its modulation of central or peripheral vascular tone is a logical starting point.

Cardiovascular autonomic dysfunction is gaining attention as a secondary and transient consequence of concussion with empirical evidence (4, 5, 7–11) and topical reviews (12, 13), demonstrating a vast array of the dysfunction’s manifestation. The empirical evidence demonstrates the presence of a transient autonomic dysfunction from digital recordings of the electrocardiogram (ECG) (4, 5, 8–10). Permutations of the ECG and HR signal provide indirect measurements of variability to sinoatrial pacing (14) and myocardial repolarization lability (15) that are accepted to represent the predominance of parasympathetic modulation at rest, and, less reliably (i.e., in terms of intra and inter-visit reproducibility), of sympathetic outflow during most states of elevated psychophysiological stress (16).

An alternative indirect approach to evaluate sympathetic nervous system (SNS) control is through arterial stiffness and characterizing the contours of the arterial pulse wave (APW) (17). The normal APW has two characteristic peaks: that of systole (early), and a reflected wave from the periphery, which occurs variably during the descent to diastole (18). Increasing degrees of arterial stiffness from augmentation of peripheral SNS vasomotor tone (via acute changes to psychophysiological state or chronic adaptation to morbidity), which serves to decrease lumen diameter and increase peripheral vascular resistance above basal tone, alters the magnitude and latency of the reflected wave (19). It remains unclear how concussion injuries influence SNS control of the cardiovascular system, let alone characteristics of the APW. The present study investigated whether a concussive head injury adversely affected the contours of the peripheral APW compared to controls at rest and during an isometric handgrip test (IHGT) within 48 h of concussive injury, and again, 1 week later. An exploratory analysis sought to explore how the duration of time until RTP following concussion influenced the peripheral arterial pulse contour in concussed athletes.

## Materials and Methods

### Subjects

Ten intercollegiate athletes with a sports-related concussion and seven demographically matched (i.e., sex, age, height, body mass) and, when possible, sport position-matched (i.e., assumption of similar fitness) control subjects voluntarily participated. A player suspected of sustaining a head injury was identified by the sports medicine staff (i.e., certified athletic trainers and team physician, on the field or in the clinic) and removed from athletic activity for further independent-clinical evaluation and confirmation using accepted practices of concussion assessment and management. Clinical decision making on RTP of concussed athletes was made by a certified athletic trainer and team physician, independent of any input from the research evaluation. As part of a secondary screening by the research investigators to verify capacity at the time of enrollment/study, the concussed athlete was required to demonstrate the ability to orient to person, place, time, and a current event to ensure that an informed decision was achieved. All participants provided written informed consent and the study procedures were approved by the university institutional review board. To be considered eligible for the study, the concussed athlete must: have sustained a concussion, or the presentation of initial symptoms occurred within the previous 48 h; be able to demonstrate capacity to provide written informed consent; not be taking medications with direct or indirect actions on the cardiovascular (i.e., ACE inhibitors, α-agonists or β-blockers, calcium channel blockers, etc.) or central nervous system (i.e., antidepressants, anti-epileptics, narcotics, opioids, etc.); be free of acute illness or trauma that would otherwise preclude their involvement in the study. Control subjects were recruited and enrolled based on meeting the previously outlined guidelines, excepting for the presence of concussion.

### Data Collection and Signal Processing

The initial study observation was completed 48 h after the concussion, or the clinical presentation of concussive symptoms occurred. The identical procedures were repeated 1 week after the initial study visit. To offset contamination of SNS outcomes by circadian variability, data collection occurred between 8:00 and 11:30 a.m. for all study visits; efforts were made to begin the second study visit within 30 min of the time of the initial visit. Upon arrival on the respective test dates, all subjects were asked a series of questions pertaining to the injury and underwent a review of symptoms and examination to verify the presence of concussion (in the concussion group). At each study visit, a continuous digital 3-lead ECG (Lead II) and beat-to-beat finger arterial blood pressure using photoplethysmography (20) was collected during the respective rest (i.e., 5 min of quiescent seating) and IHGT study procedures. After instrumentation and before data collection, participants remained seated in the upright position with their right arm elevated and resting on a foam pad at heart level (and parallel to the surface of the floor) with the elbow flexed to 90^o^ at quiet rest for approximately 20 min to acclimate to the testing facility. After acclimation to the seated position, 5 min of continuous digital resting data were obtained during which the subjects were instructed to breathe at their self-selected pace. For the IHGT, subjects were instructed to perform two maximal voluntary contractions (MVC) with the left hand on a handgrip dynamometer (21, 22). The values from the two MVC attempts were averaged and the participant was asked to perform a 3-min IHGT at 30% of MVC. The MVC from the initial study was repeated at the 1-week follow-up visit for consistency of the provocation. For the purposes of data presentation, the first 60 s (F60) of the IHGT were analyzed because this is when autonomic reciprocity (i.e., vagal nerve activity is withdrawn from the sinoatrial node and SNS activity increases) accommodates the test condition and is the most pronounced. ANS control of the cardiovascular system during the latter stages of the IHGT is driven by the metaboreflex and may not fully demonstrate the nature and presence of the dysfunction.

Electrocardiogram site preparation was performed according to clinical standards (23) and the placement of three electrodes was standardized (modified limb leads placed distal to the mid-clavicle bilaterally and precordial lead V5) for continuous HR monitoring. Beat-to-beat finger arterial pressure was obtained from the second and third fingers of the right hand using a commercially available and FDA-approved photoplethysmography-based blood pressure monitor (CNAP Monitor 500, CNSystems Medizintechnik AG, Graz, Austria); the signal output was digitized (NIBP100D, Biopac Systems Inc., Goleta, CA, USA) and integrated into the continuous data record. All digital ECG and beat-to-beat blood pressure data were collected at a sampling rate of 500 Hz with a commercially available data acquisition system (Biopac MP150, Biopac Systems Inc., Goleta, CA, USA) using a proprietary software (Acknowledge v. 4.2, Biopac Systems Inc., Goleta, CA, USA). Data were archived on a computer hard drive for subsequent offline analysis. Finger arterial pressure was calibrated by the device for each data collection against the brachial artery pressure.

**Figure 1:**
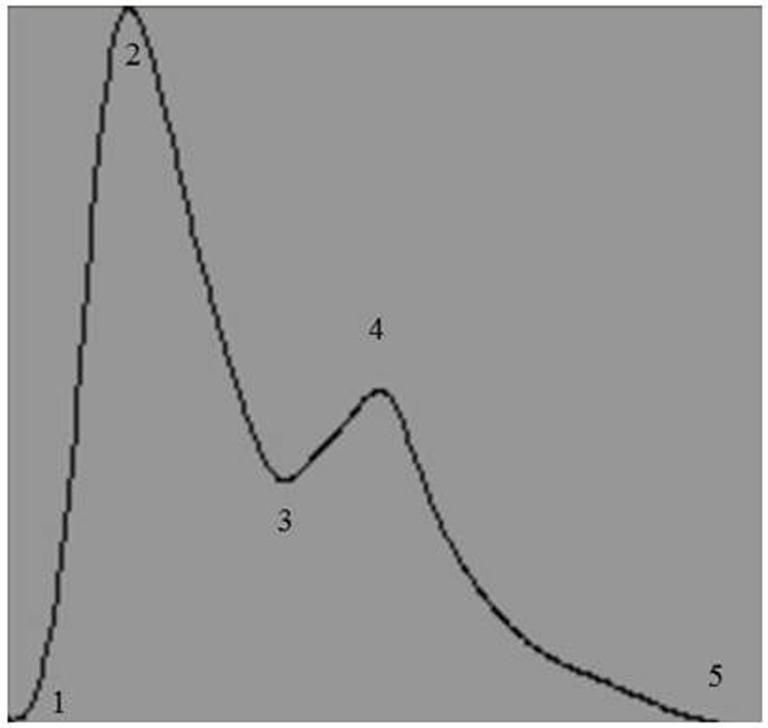
**Representative output of the composite arterial pulse wave obtained after analysis by a customized arterial pulse contour analysis program**. Numbers represent the temporal events identified by investigators: 1, start of systole; 2, peak systolic pressure; 3, dicrotic notch pressure; 4, peak pressure of the reflected wave; and 5, end of pulse wave.

**Figure 2:**
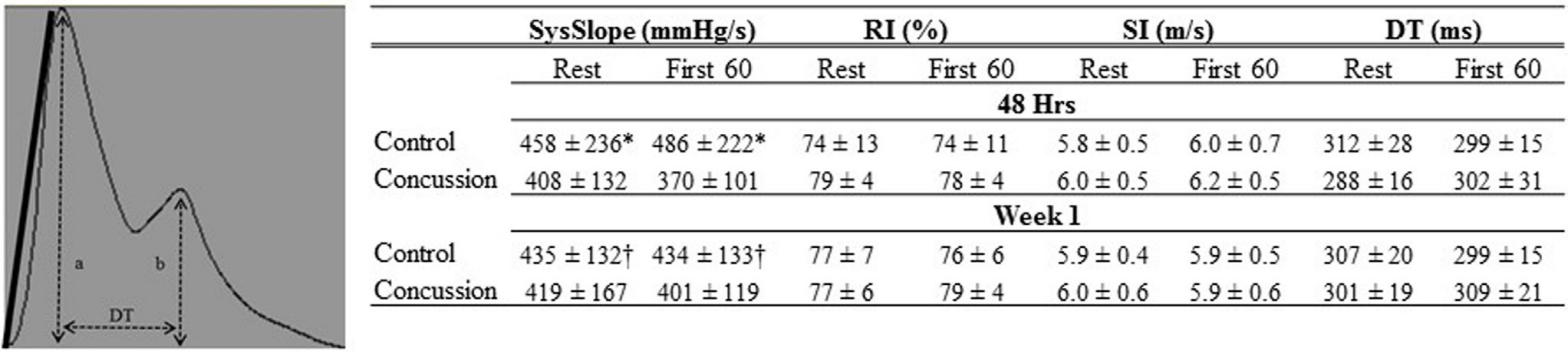
**Finger arterial pulse contour characteristics of groups by condition and visit**. The Systolic slope (SysSlope; black line, left panel) is calculated from the rate of rise (change in pressure/change in time) of the systolic upstroke. The reflection index (RI) is calculated as b/a. The stiffness index (SI) is calculated as body height/DT. Data are presented as group mean ± SD in table (right panel). *Control vs. concussion: *p* < 0.0001; †control vs. concussion: *p* < 0.01.

**Figure 3:**
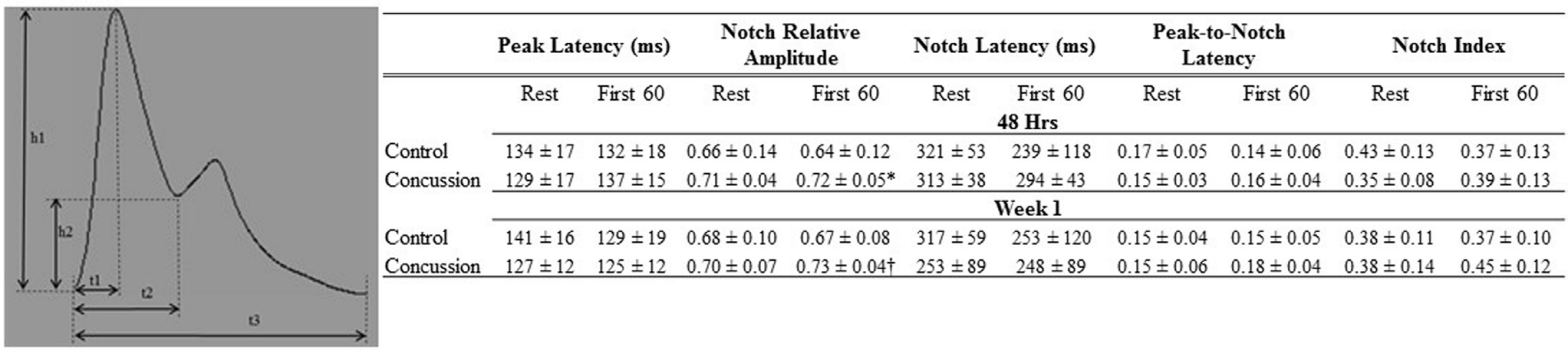
**Dicrotic notch characteristics of groups by condition and visit**. Schematic of dicrotic notch parameters (left panel) for pulse (h1) and notch (h2) amplitudes, as well as time to peak (t1), notch time (t2), and pulse time (t3). Data are presented as group mean ± SD in table (top panel). *Control vs. concussion: *p* = 0.07; †control vs. concussion: *p* = 0.09. These events were used to calculate: peak latency (t1/t3), notch relative amplitude (h2/h1), notch latency (t2/t3), peak-to-notch latency [(t2−t1)/t3)], and notch index [t2/(t3−t2)].

Raw digital data files were filtered with a zero-lag fourth-order Butterworth filter with default cut off frequencies of 6 Hz (high pass) and 100 Hz (low pass filter). The signals were visually inspected for artifact and ectopic beats to create a representative R–R interval time series. For each data file, APWs were visually inspected and ectopic or distorted beats were removed from the analysis using a customized LabView program (National Instruments, Austin, TX, USA). To characterize the APW contours, investigators inspected each non-distorted waveform to identify the start of systole, peak systolic pressure, the dicrotic notch, the peak diastolic deflection, and the end diastolic pressure (Figure 1). Once all valid waveforms were annotated in the data file, a composite arterial waveform was generated with the pressure amplitude and event latencies identified. From these characteristics, the systolic slope (i.e., change in pressure divided by the change in time from the systolic upstroke), and reflection index (RI; peak pressure of the reflected wave divided by the peak systolic pressure multiplied by 100) and stiffness index (SI; participant body height divided by the time difference between peak systolic and reflected wave pressure (DT) were characterized (Figure 2, left panel) (24, 25). In addition, parameters from the dicrotic notch were identified to characterize (Figure 3, bottom left panel) temporal features of the APW (26).

### Blood Draw and Analyses

At each visit and upon completion of hemodynamic assessments, venous blood samples were obtained from the antecubital vein in a subset of participants (i.e., control = 4 and concussion = 5). Upon collection, blood samples were spun and separated according to standard clinical and laboratory practices. The remaining serum was immediately stored frozen until being sent to a commercial laboratory for analysis (Quest Diagnostics, Teterbobo, NJ, USA). Serum electrolyte concentrations for sodium, potassium, chloride, and osmolality were determined and compared against the normal reference range for the respective marker; these blood markers were used to rule out the presence of electrolyte deficiencies or dehydration that may confound the interpretation of pulse wave indices.

### Statistics

Values are expressed as group mean ± SD. Factorial analysis of variance (ANOVA) was performed to identify group differences (control compared to concussion) for demographic (age, height, weight, BMI, DOI), hemodynamic [i.e., (HR), systolic blood pressure (SBP), diastolic blood pressure (DBP), mean arterial pressure (MAP), and pulse pressure (PP)], and pulse contour (i.e., SysSlope, RI, SI, DT) and dicrotic notch characteristics (peak latency, notch relative amplitude, notch latency, peak-to-notch latency, and notch index) for each condition (i.e., rest and during the F60 of the IHGT) and study visit (i.e., 48 h, and Week 1). Factorial ANOVA was performed to identify group differences in serum electrolyte concentrations at each study visit (i.e., 48 h and Week 1). When significant group differences were identified within a condition or visit, unpaired *t*-tests were performed to characterize whether the direction or magnitude of change was different between groups for the respective outcome. For exploratory and descriptive purposes, linear regression analyses were performed on study outcomes that were identified as being statistically significant by factorial ANOVA, or those that could provide mechanistic insight into the transient pathophysiological state. These analyses included comparisons between the control and primary concussion group and also with a further subdivision of the concussion group based on the independent-clinical determination of RTP such that two separate cohorts were formed (i.e., RTP ≤ 1week and RTP > 1week). In other words, specific resting characteristics for each group from the 48 h time point were plotted and analyzed to determine if an inference of injury severity, as determined by the duration until RTP, resulted in the ability to further differentiate the group with concussion from controls by arterial pulse contour outcomes. An *a priori* level of significance was set at *p* ≤ 0.05. Statistical analyses were completed using IBM SPSS Statistics 21 (IBM, Armonk, NY, USA).

## Results

Demographic and hemodynamic data for subjects in the concussion and control groups are provided (Tables 1 and 2, respectively). The groups were well matched for demographic characteristics and did not differ hemodynamically between conditions across the study visits. No athlete reported experiencing pain conditions that may otherwise contaminate the study outcomes. Serum electrolyte concentrations did not differ between groups at the study visits and all laboratory values were within the normal range of the respective marker (Table 3). Finger arterial pulse contour characteristics (i.e., RI, SI, and DT) were not different between the groups (i.e., concussion compared to control) among conditions or between study visits (Figure 2). SysSlope was significantly lower in the concussion group at rest and during the F60 of the IHGT at 48 h and 1 week post-injury. Secondary analysis revealed that the concussion group had a paradoxical decline in SysSlope at each visit during the transition from rest to IHGT, with the magnitude of the decline being greater at the 48 h study visit (Figure 2). Dicrotic notch characteristics were largely unremarkable between the groups at the respective study time points and conditions, with exception for a trend toward a significant elevation in the notch relative amplitude among concussed athletes during the F60 of the IHGT (Figure 3). Exploratory analyses revealed a highly significant positive correlation between resting PP and SysSlope in both the concussion (*r*^2^ = 0.84, *p* < 0.0001) and control (*r*^2^ = 0.97, *p* < 0.0001) groups at 48 h (Week 1 data was also significantly correlated, but is not shown).

**Table 1:** **Group demographics**.

	Control	Concussion
*n*	7	10
Age (year)	20.0 ± 1.4	19.4 ± 1.1
Height (m)	1.80 ± 0.11	1.79 ± 0.11
Weight (m)	81.2 ± 13.8	78.4 ± 12.2
BMI (kg/m^2^)	24.8 ± 1.7	24.7 ± 4.0
Gender (M/F)	6/1	8/2

*Data are presented as group mean ± SD*.

**Table 2:** **Hemodynamic Characteristics of Participants**.

	HR (bpm)		SBP (mmHg)		DBP (mmHg)		MAP (mmHg)		PP (mmHg)	
	Rest	First 60	Rest	First 60	Rest	First 60	Rest	First 60	Rest	First 60
**48 H**										
Control	68 ± 7	67 ± 4	120 ± 25	126 ± 28	59 ± 5	61 ± 9	80 ± 9	82 ± 12	61 ± 27	66 ± 27
Concussion	63 ± 10	67 ± 11	119 ± 19	119 ± 20	64 ± 9	64 ± 13	83 ± 12	82 ± 14	55 ± 13	54 ± 12
**Week 1**										
Control	62 ± 9	64 ± 8	118 ± 21	119 ± 22	57 ± 10	62 ± 16	77 ± 12	81 ± 17	61 ± 19	56 ± 11
Concussion	69 ± 10	73 ± 9	121 ± 15	122 ± 15	67 ± 11	71 ± 8	85 ± 11	88 ± 9	54 ± 12	51 ± 13

*Data are presented as group mean ± SD. HR, heart rate; SBP, systolic blood pressure; DBP, diastolic blood pressure; MAP, mean arterial pressure; PP, pulse pressure*.

**Table 3:** **Serum electrolyte concentrations by group**.

Laboratory	Normal Range	Group	48 h	Week 1
Na + (mmol/1)	135–146	Control	139 ± 0.0	140 ± 1.0
		Concussion	141 ± 0.8	140 ± 2
K + (mmol/1)	3.5–5.3	Control	4.5 ± 0.4	4.5 ± 0.3
		Concussion	4.4 ± 0.3	4.2 ± 0.2
CI− (mmol/1)	98–110	Control	104 ± 1.3	104 ± 2.2
		Concussion	104 ± 3.1	104 ± 3
Osm (mOsm/kg)	275–305	Control	286 ± 2	284 ± 4
		Concussion	281 ± 4	281 ± 5

*Data are presented as group mean ± SD. Sample size per group: control = 4; concussion = 5*.

With further separation of the concussion group into two separate cohorts based on the independent-clinical determination of RTP, six athletes with concussion were removed from participation for <1 week (i.e., RTP ≤ 1week) and four were exempt from participation for more than 1 week (i.e., RTP > 1week). For descriptive purposes, the RTP > 1week group had lower mean values compared to the RTP ≤ 1week and control group in the SysSlope (405 ± 200; 420 ± 88; 454 ± 236 mmHg/s, respectively), peak-to-notch latency (0.12 ± 0.04; 0.16 ± 0.02; 0.17 ± 0.05, respectively), notch relative amplitude (0.70 ± 0.03; 0.71 ± 0.04; 0.66 ± 0.14, respectively), and PP (54 ± 16; 56 ± 13; 61 ± 27, respectively). The RTP > 1week group had an elevated mean SI compared to the RTP ≤ 1week and control group that approached statistical significance (6.4 ± 0.2; 5.7 ± 0.4; 5.8 ± 0.5, respectively). Secondary analysis revealed that the SI and peak-to-notch latency approached a significant linear relationship (*r*^2^ = 0.88, *p* = 0.06) in the RTP > 1week group, but not the others. Similarly, a significant linear relationship (*r*^2^ = 0.70, *p* < 0.05) between the SI and notch relative amplitude was observed in the RTP ≤ 1week group, but not the others. The relationship in the RTP > 1week group remained in the corresponding analysis of week 1 data, suggesting a persisting disturbance (*r*^2^ = 0.79, *p* = 0.06, data not shown), whereas the relationship in the RTP ≤ 1week group had resolved (*r*^2^ = 0.04, *p* = NS, data not shown).

## Discussion

The current report is the first to examine and characterize the changes to finger arterial pulse contours and their latencies at rest and during a low-intensity provocation in the first post-injury week following concussion. Our findings demonstrate that subtle, but meaningful differences emerged in the APW to differentiate those who sustained a concussion from a non-concussed cohort. The changes to these outcomes were more disparate in those concussed individuals who had a longer duration until RTP, from those with a shorter RTP and controls. This suggests that the clinical severity of concussion resulted in a more adverse APW profile (i.e., assumption herein is that length of time until RTP is a reflection of the clinical severity of the injury). The most prominent mechanistic finding from this report is that the SysSlope, which was significantly correlated to the arterial PP, was reduced in concussed athletes and paradoxically responsive to a low-intensity provocation (i.e., IHGT) 48 h after concussion; this dysfunction became more apparent after separating those concussed athletes who did not RTP for more than 1 week after injury from those who resumed play within 1 week.

The arterial PP represents the difference between systolic and diastolic blood pressure. The PP is correlated to indices of left ventricular blood flow dynamics, including ejection fraction (27) and stroke volume (28). The presence of a low (29) or elevated (30–32) PP in clinical cohorts with cardiovascular morbidity has served as a strong predictor for future incident cardiac events, including death. One is then able to surmise that the PP is a reflection of left ventricular function across the spectrum of cardiovascular health and disease. Our present findings for a strong linear association between PP and SysSlope would then suggest that changes to the latter are a manifestation of left ventricular (dys)function, and more specifically stroke volume. Thus, the observed paradoxical decline in SysSlope during the transition from rest to test with the IHGT among concussed athletes at 48 h may be the result of reduced stroke volume. In order to *maintain* cardiac output, changes to stroke volume must be met by a reciprocal compensation in HR. This was the case in our concussed cohort, as HRs increased from rest to test with the associated and inferred decline in stroke volume. The dysfunction in the concussion group emerges when comparing the observed responses and transition from rest to test in our control group who had an increase in SysSlope (i.e., stroke volume) and no appreciable change in HRs. The control group response would result in an *increase* cardiac output, which is expected when a systemic provocation increases metabolic demand, even with a low-intensity provocation, such as the IHGT (21, 22). Therefore, we speculate that a component of post-concussive exercise intolerance may be from reduced cardiac sympathoexcitation resulting in insufficient left ventricular inotropism that leads to lower stroke volumes.

It is generally accepted that the latency and amplitude of the reflected wave in the arterial pulse is the product of peripheral arterial stiffness (19). By quantifying the latency and amplitude between cohorts (i.e., concussion and control) and conditions (i.e., rest and IHGT), our results demonstrated that the post-­concussive sequela includes an abnormal element of arterial stiffness within the first post-injury week. The SI, which was calculated from the time between the occurrence of the peak systolic and reflected wave, differentiated the RTP > 1week group from the RTP ≤ 1week and control groups. The nature of this dysfunction emerged through several different features of the APW. The notch relative amplitude (i.e., the pressure amplitude of the dicrotic notch relative to peak systolic pressure) was elevated among concussed participants during the F60 of the IHGT at each visit; the simplest interpretation of this outcome is that the dicrotic notch occurred at an elevated pressure in the concussion cohort compared to controls. The peak-to-notch latency (i.e., the time between peak systole and dicrotic notch, relative to the time of the pulse duration) was unremarkable between primary groups (i.e., concussion and control), but when separating the concussion group by RTP, the RTP > 1week cohort was dramatically shorter compared to the other cohorts. The relative timing of the dicrotic and its pressure excursion to a relatively elevated pressure would be an anticipated result of increased peripheral arterial stiffness. In an attempt to reconcile the relationship between the SI and notch pressure-latency characteristics in the concussion cohorts, a differential linearity emerged in the RTP sub-groups. In the RTP ≤ 1week cohort, the notch relative amplitude was significantly related to SI, but had no association in the other cohorts. In the RTP > 1week cohort, the peak-to-notch latency approached a statistically significant linear relationship to SI, and like before, was not associated in other groups. Thus, the characteristics of peripheral arterial stiffness appear to manifest differently in athletes with concussion who RTP within 1 week from those who do not resume participation until after 1 week. If confirmed by a larger study, this finding has the potential to facilitate a differential “diagnosis” on the severity of concussion and perhaps, crudely predict the length of time until RTP. The presence of increased peripheral artery stiffness following concussion could be speculated to be a compensation strategy to obviate the fall in stroke volume that was previously described.

A puzzle emerges when attempting to reconcile the presumed effects of SNS modulation at the heart compared to the peripheral arteries in the concussed cohort. The finding of a paradoxical response in SysSlope, an indirect surrogate of ventricular function, and perhaps, stroke volume, during the transition from rest to test would result from a dearth of myocardial sympathoexcitation. Conversely, the presence of increased peripheral arterial stiffness would suggest augmented SNS activity. Thus, central and peripheral autonomic integration is adversely affected in a transient manner to concussion. This confounded autonomic integration may contribute to the emergence of exercise intolerance in the first post-injury week, which could manifest as inappropriate hemodynamic responses and contribute to a reduced capacity to maintain or achieve varying intensities of work. It is then expected that those individuals in whom recovery has not yet occurred will be less likely to accommodate changes in systemic metabolic demand by recruiting appropriate cardiovascular responses through the ANS. The premise of autonomic dissociation following brain injury is not new, as Goldstein and colleagues previously demonstrated that the severity of neurological (brain) injury was related to the degree of uncoupling of cardiovascular autonomic control (33). In other words, the ability of the ANS to regulate cardiac (i.e., chronotropic and inotropic) and vasomotor outflow in response to hemodynamic perturbations is confounded across the spectra of brain injury leading to a complete dissociation at brain death (33).

### Limitations

There were some limitations in our study. First, our sample size was small and may not be generalizable to a larger audience. To enhance the descriptive nature of the study, we separated the concussion group into two cohorts based on the duration of time until RTP; this categorization was then used as a surrogate injury severity to further our interpretation. We appreciate that this is not a “gold-standard” approach. Blood samples were collected from a subset of participants in the trial; we approximated that the lack of abnormal lab values was characteristic of the entire cohort. The most frequent barrier to obtaining a blood sample arose from a fear/dislike of needles among participants; this fear may also pose as a barrier to future exploration in the field derived from specimen. As such, non-invasive measurements that are found to be sufficiently reliable to physiological changes may hold the most promise for immediate exploration. The IHGT was used to provoke the ANS system. This is a validated, low-intensity challenge that can safely be performed.

## Conclusion

Use of arterial pulse contour analyses revealed that concussed athletes have a transient increase in peripheral artery stiffness, which may be a compensatory adaptation to a paradoxical decline of SysSlope, an indirect measurement of stroke volume, during the transition from rest to a state of increased metabolic demand. Dysfunction of the SNS appeared to be more pronounced among concussed athletes who were removed from athletic participation for more than 1 week compared to those who resumed play within 7 days. Further work is needed to determine how and when this dysfunction resolves over a 10- to 14-day period; whether or not it persists among individuals whose symptoms do not resolve and the injury becomes a chronic morbidity; and how the variables respond to more “intense” provocations that may be used during the evaluation of a progressive RTP.

## Author Contributions

ML designed the study, collected data, performed statistical analyses, and completed the manuscript. MT assisted in the screening, recruitment and clinical care of participants, and reviewed final draft of manuscript. AT assisted in the screening, recruitment and clinical care of participants, and reviewed final draft of manuscript. VH-L assisted with data analysis and review of final draft of manuscript.

## Conflict of Interest Statement

The authors declare that the research was conducted in the absence of any commercial or financial relationships that could be construed as a potential conflict of interest.
